# Induced hypothermia of 33°C does not affect host response compared with maintaining 36°C

**DOI:** 10.1186/cc13686

**Published:** 2014-03-17

**Authors:** C Beurskens, J Horn, E Van Leeuwen, N Juffermans

**Affiliations:** 1 AMC, Amsterdam, the Netherlands

## Introduction

Induced hypothermia is applied in the ICU and in the operating theater to reduce ischemia-reperfusion injury. It is thought that induced hypothermia may hamper the immune response and therefore carry the risk of acquiring or aggravating an infection. We investigated the effect of hypothermia on host response by comparing survivors of cardiac arrest in which body temperature was kept at either 33°C or 36°C.

## Methods

As a substudy to the Target Temperature Management trial [1] in which cardiac arrest patients admitted to the ICU were randomized to maintaining either 33°C (*n *= 11) or 36°C (*n *= 9) for 24 hours, blood was drawn at the start and end of the target temperature phase as well as after reaching normotemperature. Host response was measured via monocyte human leukocyte antigen-DR (HLA-DR) expression and via whole blood stimulation with TLR ligands lipopolysaccharide (LPS) and lipoteicoic acid (LTA) for 24 hours. Plasma levels of interleukin (IL)-1β, IL-1RA, IL-6, IL-8, IL-10, tumor necrosis factor alpha (TNFα), macrophage inflammatory proteins (MIP)-1, monocyte chemotactic protein (MCP)-1 and soluble CD40 ligand levels were determined with ELISA or Luminex. Statistics were by unpaired Mann-Whitney *U *tests.

## Results

HLA-DR expression was decreased compared with healthy controls, without differences between 33°C and 36°C. Following whole blood stimulation with LPS, TNFα and IL-6 production was lower after cardiac arrest compared with healthy controls. The 33°C and 36°C groups differed at baseline in TNFα levels after LPS whole blood stimulation. After 24 hours of temperature management, there was no difference in both TNFα and IL-6 production between the groups following TLR ligand stimulation. After cessation of temperature management only TNFα levels increased after LTA stimulation. Plasma levels of IL-1RA, IL-8 and IL-10 were decreased after cardiac arrest compared with healthy controls. In plasma levels of IL-1β, MIP-1 and soluble CD40 ligand there were no differences between the 33°C and 36°C groups at all time points. Apart from baseline differences, there were no differences between 33°C and 36°C in plasma levels of IL-1RA, IL-8, IL-10 and MCP-1.

## Conclusion

Cardiac arrest induces a decrease in proinflammatory response. There were no differences in immune host response after 24 hours of targeting body temperature at 33°C or 36°C. These results suggest that hypothermia does not alter host immune response.
